# Serum Amino Acids in Association with Prevalent and Incident Type 2 Diabetes in A Chinese Population

**DOI:** 10.3390/metabo9010014

**Published:** 2019-01-14

**Authors:** Yonghai Lu, Yeli Wang, Xu Liang, Li Zou, Choon Nam Ong, Jian-Min Yuan, Woon-Puay Koh, An Pan

**Affiliations:** 1 Saw Swee Hock School of Public Health, National University of Singapore, Singapore 117549, Singapore; ephluy@nus.edu.sg (Y.L.); danfordwyl@gmail.com (Y.W.); ephzouli@nus.edu.sg (L.Z.); woonpuay.koh@duke-nus.edu.sg (W.-P.K.); 2 Institute of Nutrition and Health, Qingdao University, Qingdao 266071, China; 3 NUS Environmental Research Institute, National University of Singapore, Singapore 117411, Singapore; eriliaxu@nus.edu.sg; 4 Division of Cancer Control and Population Sciences, University of Pittsburgh Cancer Institute, Pittsburgh, PA 15232, USA; yuanj@upmc.edu; 5 Department of Epidemiology, Graduate School of Public Health, University of Pittsburgh, Pittsburgh, PA 15232, USA; 6 Health Services and Systems Research, Duke-NUS Medical School Singapore, Singapore 169857, Singapore; 7 Department of Epidemiology and Biostatistics, School of Public Health, Tongji Medical College, Huazhong University of Science and Technology, Wuhan 430030, China; 8 Ministry of Education Key Laboratory of Environment and Health and State Key Laboratory of Environmental Health (incubation), School of Public Health, Tongji Medical College, Huazhong University of Science and Technology, Wuhan 430030, China

**Keywords:** Essential amino acids, non-essential amino acids, type 2 diabetes, serum, LC-MS/MS

## Abstract

We aimed to simultaneously examine the associations of both essential and non-essential amino acids with both prevalent and incident type 2 diabetes in a Chinese population. A case-control study was nested within the Singapore Chinese Health Study. Participants included 144 cases with prevalent and 160 cases with incident type 2 diabetes and 304 controls. Cases and controls were individually matched on age, sex, and date of blood collection. Baseline serum levels of 9 essential and 10 non-essential amino acids were measured using liquid chromatography tandem mass spectrometry. We identified that five essential (isoleucine, leucine, lysine, phenylalanine, and valine) and five non-essential (alanine, glutamic acid, glutamine, glycine, and tyrosine) amino acids were associated with the prevalence of type 2 diabetes; four essential (isoleucine, leucine, tryptophan, and valine) and two non-essential (glutamine and tyrosine) amino acids were associated with the incidence of type 2 diabetes. Of these, valine and tyrosine independently led to a significant improvement in risk prediction of incident type 2 diabetes. This study demonstrates that both essential and non-essential amino acids were associated with the risk for prevalent and incident type 2 diabetes, and the findings could aid in diabetes risk assessment in this Chinese population.

## 1. Introduction

The global prevalence of diabetes among adults is soaring, rising from 4.7% in 1980 to 8.5% in 2014 [1]. Type 2 diabetes comprises 90–95% of diabetes, and is characterized by high blood glucose, insulin resistance, and relative insulin deficiency [2]. The pathogenesis of type 2 diabetes is complex and involves interaction of genetic and environmental influences. Several epidemiologic studies using metabolomics strategies have reported that the increase of certain essential amino acids such as branched-chain amino acids (BCAAs) and aromatic amino acids (AAAs) was associated with a high risk of type 2 diabetes in Western and Asian populations [3,4,5,6,7,8]. Further, Tilin et al. [9] and Lee et al. [10] reported ethnical differences in blood levels of BCAAs and AAAs and suggested that these differences may add explanatory insights into the increased risk of diabetes in multiethnic cohorts. These observations provide a new angle on the pathogenesis of type 2 diabetes, thus attracting worldwide attention. However, recent studies have shown conflicting data on the effects of BCAAs on glucose metabolism and insulin resistance [11,12,13]. In a recent prospective study, Zheng et al. reported that high intake of BCAAs is associated with an increased risk of type 2 diabetes in three large cohorts of US women and men [11], while one study conducted in a Japanese population found that a high intake of BCAAs is associated with a low risk of type 2 diabetes [13]. Compared to the essential amino acids, non-essential amino acids can be produced in the body and are less influenced by the dietary factors, and their concentrations may be more related to the metabolic disorders in type 2 diabetes. In this study, we thus aimed to simultaneously examine and verify the relation of both essential and non-essential amino acids with both prevalent and incident type 2 diabetes in a population-based cohort study, the Singapore Chinese Health Study (SCHS).

## 2. Results

### 2.1. Method Validation

A total of 19 amino acids, including 9 essential (histidine, isoleucine, leucine, lysine, methionine, phenylalanine, threonine, tryptophan, and valine) and 10 non-essential amino acids (alanine, arginine, asparagine, aspartic acid, glutamic acid, glutamine, glycine, proline, serine, and tyrosine), were quantified in this study (Supplementary Table S1). The proposed analytical method was validated for limit of detection (LOD), linearity, recovery, accuracy, and precision (Supplementary Table S2).

### 2.2. Participant Characteristics

Table 1 shows baseline characteristics of the participants, in which prevalent and incident type 2 diabetes were presented separately. In this study, we divided the cases into prevalent and incident type 2 diabetes cases according to their baseline HbA1c levels: prevalent cases with HbA1c ≥ 6.5% (47.5 mmol/mol) and incident cases with HbA1c < 6.5% (47.5 mmol/mol). Cases with prevalent and incident type 2 diabetes had higher levels of random glucose, BMI, triglycerides and lower levels of high-density lipoprotein (HDL)-cholesterol than controls at baseline (*p* ≤ 0.001), and they were also more likely to report a history of hypertension. As noted, 27.3% participants (166 out of 608) were fasted before blood collection.

### 2.3. Associations Between Amino Acids and Type 2 Diabetes

Associations between amino acids and the risk for prevalent type 2 diabetes are shown in Table 2. After adjustment for confounders, including BMI, history of hypertension, smoking status, HDL-cholesterol, and triglycerides, 10 amino acids were associated with prevalent type 2 diabetes at *p* < 0.05, including 5 essential (isoleucine, leucine, lysine, phenylalanine, and valine) and 5 non-essential (alanine, glutamic acid, glutamine, glycine, and tyrosine) amino acids. All the 10 amino acids were significant with an FDR cut-off of 0.1. Although 0.05 is commonly used as a cutoff limit for FDR in the discovery of metabolic signatures, the metabolites with FDR between 0.05 and 0.1 are still considered marginally significant. To avoid missing potential biomarker candidates associated with risk for type 2 diabetes, we applied 0.1 as the cutoff value for FDR. The associations between amino acids and the risk for incident type 2 diabetes were shown in Supplementary Table S3. After adjustment for BMI, history of hypertension, smoking status, HDL-cholesterol, and triglycerides, 6 amino acids were associated with incident type 2 diabetes at *p* < 0.05, including 4 essential (isoleucine, leucine, tryptophan, and valine) and 2 non-essential (glutamine and tyrosine) amino acids. However, none of the 6 amino acids were significant at FDR < 0.1. Of these, five differential amino acids were common to prevalent and incident type 2 diabetes, including glutamic acid, tyrosine, and three BCAAs (leucine, isoleucine, and valine). The associations of 5 common amino acids with prevalent type 2 diabetes were stronger than those with incident type 2 diabetes, except tyrosine, for which the association with incident type 2 diabetes was more evident.

In our study, certain amino acid changes due to fasting status were observed (Supplementary Table S4). However, stratification by fasting status showed that fasting had no effect on the associations between amino acids with prevalent and incident type 2 diabetes (*p* for interaction > 0.5) (Supplementary Tables S5 and S6). This is possibly because the proportion of type 2 diabetes cases was equally distributed among fasting and non-fasting participants (*p* > 0.05) (Table 1). They are the reasons why we did not include fasting status in the models. In contrast, we reasoned that smoking is an important risk factor for type 2 diabetes and thus included it in the models.

### 2.4. Predictive Value of Amino Acids for Incident Type 2 Diabetes

We evaluated the predictive performance of amino acids for the risk of incident type 2 diabetes using c- statistics, and IDI and NRI indexes (Table 3). Adding the 19 amino acids individually did not significantly improve AUCROC values compared to base model of BMI, history of hypertension, smoking status, HDL-cholesterol, and triglycerides, whereas IDI and NRI statistics showed that valine and tyrosine could improve risk reclassification of incident type 2 diabetes at *p* < 0.05. In addition to individual amino acid, we explored the predictive performance of certain amino acid combinations through binary logistic regression, including combinations of 6 amino acids associated with incident type 2 diabetes (combination 1), 9 essential amino acids (combination 2), 10 non-essential amino acids (combination 3), and all the 19 amino acids (combination 4). We found that all the 4 combinations could significantly improve risk reclassification of incident type 2 diabetes with a *p* < 0.01 in IDI and NRI statistics, yet only the combination 4 led to a significant increase in area under the receiver operating characteristic curve (AUCROC) value (*p* = 0.04).

## 3. Discussion

In this case-control study, we simultaneously quantified 9 essential and 10 non-essential amino acids using LC-MS/MS. The increase of 6 essential (isoleucine, leucine, lysine, phenylalanine, tryptophan, and valine) and 3 non-essential (alanine, glutamic acid, and tyrosine) amino acids was associated with a higher risk for prevalent and/or incident type 2 diabetes. On the other hand, accumulation of glutamine and glycine was associated with a lower risk of type 2 diabetes. Notably, we found that these associations were influenced by random glucose levels. With further adjustment for random glucose, none of these amino acids remained significant associations with type 2 diabetes. Moreover, we examined the predictive performance of amino acids for type 2 diabetes and found that valine and tyrosine showed positive predictive value for diabetes risk in this Chinese population.

Essential amino acids such as BCAAs are mainly derived from dietary intake and are metabolized in skeletal muscle and adipose tissue [14]. Numerous epidemiologic studies have identified blood BCAAs as among the most consistent and important metabolites associated with insulin resistance [3,15,16,17]. In consistent with these studies, we found that BACCs (isoleucine, leucine, and valine) were significantly increased in prevalent and incident type 2 diabetes cases compared to controls. Although not completely understood, there exists several possible mechanisms underlying the association between BCAAs and the risk of type 2 diabetes. First, a recent study by Pedersen et al. shown that altered intestinal microbiota impacted BCAA levels and may contribute to insulin resistance [18]. Further, BCAAs can increase skeletal muscle glucose uptake and stimulate glycogen synthesis in an insulin-independent manner through phosphatidylinositol 3-kinase or protein kinase C [19]. In addition, the interplay of adipose tissue, BCAA metabolism, and glucose homeostasis was reported recently [20]. BCAAs may produce more catabolic intermediates such as propionyl CoA and succinyl CoA, leading to accumulation of incompletely oxidized fatty acids and glucose.

AAAs, particularly phenylalanine and tyrosine, have been reported to be elevated together with BCAAs in obesity-related insulin resistance [3,15,21,22]. In line with these studies, we found that phenylalanine and tyrosine were significantly increased in prevalent and/or incident type 2 diabetes cases compared to controls. In addition, tryptophan was found to be significantly increased in incident type 2 diabetes cases compared to controls in our study. Tryptophan can be metabolized to several kinds of physiologically active metabolites such as kynurenine, kynurenic acid, and serotonin via two metabolic pathways: tryptophan–kynurenine and tryptophan–methoxyindole. The changes in the two pathways have been related to several pathophysiological mechanisms of type 2 diabetes [23].

Besides well-recognized BCAAs and AAAs, we identified four non-essential amino acids (alanine, glutamic acid, glutamine, and glycine) associated with prevalent and/or incident type 2 diabetes. Among these four amino acids, alanine and glutamic acid were positively associated with risk of type 2 diabetes; glutamine and glycine were negatively associated. The finding of inverse associations between glutamine and glycine and the risk for type 2 diabetes is in line with a recent meta-analysis [16]. Alanine has recently been associated with future hyperglycemia and the risk for diabetes [24,25,26]. Glutamic acid is derived from BCAAs, catalyzed by branched-chain aminotransferase. A report from the Framingham offspring study showed that glutamic acid was positively associated with insulin resistance [27]. In this study, the increase of glutamic acid was strongly associated with the risk for prevalent and incident type 2 diabetes.

The strength of this study is its prospective design and hence the presumed lack of recall bias in exposure data prior to type 2 diabetes diagnosis. Furthermore, the roles of essential and non-essential amino acids were estimated in parallel in this study. However, several limitations in our study exist. First, more than 70% of serum samples were non-fasting, and may thus affect the metabolite levels. To address this limitation, we conducted a stratified analysis by fasting status and no significant differences were observed between fasting and non-fasting samples. Second, the levels of essential amino acids are partly influenced by the dietary habits, yet the information regarding the diet was not available for further investigation. Third, the samples size is relatively small, and the potential of amino acids for risk prediction of type 2 diabetes in Chinese need to be validated externally in a large population.

## 4. Materials and Methods

### 4.1. Chemicals and Reagents

LC-MS grade methanol, acetonitrile, formic acid, ammonium formate, and standards including amino acid standard mixture (AAS18), tryptophan, glutamine, and asparagine were purchased from Sigma-Aldrich (St. Louis, MO, USA). Isotopically labeled amino acid mix standard (MSK-A2-1.2) was purchased from Cambridge Isotope Laboratories (Tewksbury, MA, USA). Distilled water was purified “in-house” using a Milli-Q purification system (Bedford, MA, USA).

### 4.2. Study Population

The participants were from the SCHS. Detailed information of recruitment and follow-up visits for SCHS has been published previously [8,28]. Briefly, 63,257 Chinese aged 45–74 years were recruited in 1993-1998. The first follow-up visit was conducted in 1999–2004, and 32,575 participants donated the morning blood samples, in which fasting was not required. The second follow-up visit was conducted in 2006–2010. Because of the logistical and funding constraints of this study, we randomly selected 608 participants for this study, including 304 cases and 304 controls. The cases were free of diagnosed diabetes, cardiovascular disease, and cancer during the first visit when blood was collected (baseline), while reported to have type 2 diabetes during the second visit. The controls were free of aforementioned diseases both at first and second visits. The age (±3 years), sex, and date of blood collection (±6 months) of cases and controls were individually matched. The baseline serum samples were used in this study. All participants voluntarily joined this study, gave informed consents, and completed questionnaires that provided information including age, sex, demographical factors, lifestyle habits, and medical history. This study was approved by the Institutional Ethics Review Board at the National University of Singapore (ethic code: NUS-IRB code 06-027).

### 4.3. Prevalent and Incident Type 2 Diabetes: Patient Regrouping

In Singapore, diagnostic tests of diabetes at the time of two visits (1999–2004 and 2006–2010) were made according to the 1997 American Diabetes Association (ADA) criteria [29], which were based on symptoms plus random glucose concentrations, fasting glucose concentrations or 2-h glucose tolerance tests. Here, random glucose concentration presents a blood glucose measurement that was carried out either with blood taken from a fasting status, or a non-fasting status during the study visit. In 2010, HbA1c ≥ 6.5% (47.5 mmol/mol) was proposed by ADA as an additional diagnostic criterion [30]. In line with the update of diagnostic criteria, we divided the 304 cases into prevalent and incident type 2 diabetes cases according to their baseline HbA1c levels: cases with HbA1c ≥ 6.5% (47.5 mmol/mol) at baseline were defined as prevalent cases (*n* = 144); cases with HbA1c < 6.5% (47.5 mmol/mol) at baseline were classified as incident cases (*n* = 160). HbA1c values in the controls were all < 6.0% (42.1 mmol/mol).

### 4.4. Lipid Assays

Blood concentrations of-cholesterol and triglycerides were measured using colorimetric method on a chemistry analyzer (AU5800; Beckman Coulter, Brea, CA, USA).

### 4.5. Amino Acid Quantification

Serum samples (50 μL) were diluted with 50μl of distilled water containing isotope labeled amino acid mix standard (75 μM) as internal standards. The mixture was treated by 500 μL of ice-cold methanol for protein precipitation. After centrifugation at 20,817 g for 15 min at 4°C, the supernatant fraction was filtered by Thermo Scientific™ national 750µL micro-centrifugal filters (PTFE membrane, 0.2 µm pore size, non-sterile). The filtrate was dried by 99.9% purity of nitrogen (N2) and reconstituted in 200 μL of acetonitrile/water (90:10, v/v) before analysis.

Quantitative analysis of amino acids was performed on an Agilent 1200 HPLC system (Waldbronn, Germany) coupled to a 6410 Triple Quadrupole mass spectrometer (Waldbronn, Germany) equipped with an electrospray ionization source in positive ion mode. Amino acids were separated by using an Acquity UPLC BEH Amide column (2.1 mm × 100 mm, 1.7 µm, Waters). The mobile phases A (30% acetonitrile with 0.1% formic acid and 10 mmol/L ammonium formate) and B (95% acetonitrile with 0.1% formic acid and 10 mmol/L ammonium formate) were employed. The gradient program was: 0–1 min, 100% B; 1–2 min, 100–92% B; 2–10 min, 92–85% B; 10–12 min, 85–60% B; 12–14 min, 60–40% B; 14–15 min, 40–15% B; 15–19 min, 15%B; 19–19.5 min, 15–100% B. The flow rate was set at 0.5 mL/min. The column temperature and injection volume were set at 40°C and 5 μL, respectively. The mass spectrum was acquired in the multiple reaction monitoring (MRM) mode with a capillary voltage of 3500 V, gas temperature of 350°C, gas flow of 12 L/min and nebulizer nitrogen gas flow rate of 30 psi. The retention time, MRM transition, fragmentor voltage, and collision energy (CE) are shown in Supplementary Table S1.

All the 304 paired samples were analyzed blindly in 8 completely independent batches, i.e., 38 paired samples in each batch. The stable isotope labeled internal standards were used in all the sample extractions and for calibration. The quality control (QC) samples were prepared by spiking certain amount native and labeled standards into a pooled plasma from all participants, and six QCs were analyzed along with the samples in each batch to ensure the reliability of the method and the instrument stability.

### 4.6. Statistical Analysis

Demographic and clinical variables of participants were presented as mean ± standard deviation (SD) for continuous data, and proportions for categorical data. Their differences between cases and controls were tested using paired t-test for continuous variables and chi-square test for categorical variables. All the amino acids measured were detected in all of the 608 serum samples, i.e., there was no missing value. Odds ratio (OR) with 95% confidence interval (CI) and *p* value for the associations between amino acids and type 2 diabetes were calculated by conditional logistic regression models, with adjustment for confounding factors, including BMI, history of hypertension, smoking status, HDL-cholesterol, and triglycerides. The OR was represented both as tertiles and per SD increment for each amino acid, and *p* value was corrected for multiple testing via false discovery rate (FDR) using the Benjamini-Hochberg method. The correlations of amino acids with random glucose and HbA1c levels were examined in controls using Pearson partial correlation analysis, adjusting for age, sex, and BMI. The c-statistic (i.e., the area under the receiver operating characteristic curve (AUCROC)) was calculated to assess the predictive utility of each metabolite by examining the improvement in discrimination. Because of the limitation of AUCROC: it is insensitive to detect clinically important risk differences [31], we evaluated the integrated discrimination improvement (IDI) and category-free net reclassification improvement (NRI) indexes as well. Statistical analyses were performed using IBM SPSS Statistics 24 and Stata version 14.0. A two-sided *p* <0.05 or FDR <0.1 was considered as statistically significant.

## 5. Conclusions

In conclusion, this study demonstrates the previous reports of strong associations between essential amino acids, particularly BCAAs and AAAs, and the risk for prevalent and incident type 2 diabetes. Further, the associations of non-essential amino acids and the risk for prevalent and incident type 2.

## Figures and Tables

**Table 1:** Baseline characteristics of the participants.

Baseline Characteristics	Prevalent Type 2 Diabetes			Incident Type 2 Diabetes		
	Case (*n* = 144)	Control (*n* = 144)	*p* Value	Case (n = 160)	Control (*n* = 160)	*p* Value
HbA1c						
%	7.7 ± 1.6	5.6 ± 0.3		5.9 ± 0.4	5.5 ± 0.2	
mmol/mol	60.7 ± 12.6	37.7 ± 2.0		41.0 ± 2.8	36.6 ± 1.3	
Random glucose (mmol/L)	8.8 ± 4.2	4.9 ± 1.3	<0.001	5.8 ± 2.0	4.9 ± 1.2	<0.001
Age (years)	62.7 ± 6.1	62.7 ± 5.9	0.834	61.6 ± 5.6	61.9 ± 6.0	0.163
Sex, n (%)			1.000			1.000
Male	62 (43.1)	62 (43.1)		79 (49.4)	79 (49.4)	
Female	82 (56.9)	82 (56.9)		81 (50.6)	81 (50.6)	
BMI (kg/m^2^)	24.6 ± 3.6	23.1 ± 3.3	<0.001	24.6 ± 3.4	22.6 ± 3.5	<0.001
History of hypertension, n (%)			0.066			<0.001
No	84 (58.3)	99 (68.7)		75 (46.9)	118 (73.8)	
Yes	60 (41.7)	45 (31.3)		85 (53.1)	42 (26.3)	
Smoking status, n (%)			0.700			0.721
Non-smoker	99 (68.8)	102 (70.8)		106 (66.3)	109 (68.1)	
Smoker	45 (31.2)	42 (29.2)		54 (33.7)	51 (31.9)	
Alcohol consumption, n (%)			0.733			0.867
< 1 drink/week	125 (86.8)	123 (85.4)		139 (86.9)	140 (87.5)	
≥1 drink/week	19 (13.2)	21 (14.6)		21 (13.1)	20 (12.5)	
Physical activity, n (%)			0.879			0.689
<0.5 hours/week	118 (81.9)	117 (81.3)		125 (78.1)	122 (76.3)	
≥0.5 hours/week	26 (18.1)	27 (18.7)		35 (21.9)	38 (23.7)	
Education, n (%)			0.405			0.657
None	37 (25.7)	31 (21.5)		29 (18.1)	26 (16.3)	
Primary and above	107 (74.3)	113 (78.5)		131 (81.9)	134 (83.7)	
Fasting status, n (%)			0.372			0.360
Non-fasting	96 (66.7)	103 (71.5)		118 (73.8)	125 (78.1)	
Fasting	48 (33.3)	41 (28.5)		42 (26.3)	35 (21.9)	
HDL-cholesterol (mmol/L)	1.1 ± 0.2	1.2 ± 0.3	<0.001	1.1 ± 0.3	1.2 ± 0.3	<0.001
Triglycerides (mmol/L)	2.6 ± 1.9	2.0 ± 1.3	0.001	2.4 ± 1.3	1.7 ± 0.9	<0.001

Data are presented as mean ± standard deviation for continuous variables, and n (%) for categorical variables. The *p* values were calculated by paired t-test for continuous variables and Chi-square test for categorical variables.

**Table 2:** The associations between amino acids and prevalent type 2 diabetes (*n* = 144).

Amino Acids	OR Across Tertiles (T) ^a^					OR Per SD Increment ^a^		
	T1	T2, OR (95% CI)	T3, OR (95% CI)	*p* for Trend	FDR	OR (95% CI)	*p* Value	FDR
Essential amino acids								
Valine	1.00	5.00 (2.05, 12.18)	5.13 (2.05, 12.86)	0.001	0.006	1.85 (1.30, 2.64)	0.001	0.006
Leucine	1.00	2.43 (1.24, 4.73)	2.63 (1.27, 5.48)	0.007	0.033	1.55 (1.12, 2.13)	0.008	0.038
Isoleucine	1.00	2.79 (1.39, 5.62)	2.22 (1.08, 4.57)	0.026	0.068	1.34 (0.98, 1.85)	0.069	0.133
Phenylalanine	1.00	1.99 (1.03, 3.84)	2.12 (1.05, 4.26)	0.030	0.068	1.44 (1.05, 1.96)	0.022	0.073
Lysine	1.00	0.88 (0.45, 1.74)	2.28 (1.11, 4.68)	0.032	0.068	1.42 (1.04, 1.94)	0.027	0.073
Methionine	1.00	2.75 (1.39, 5.46)	1.86 (0.92, 3.80)	0.077	0.124	1.21 (0.90, 1.65)	0.209	0.265
Threonine	1.00	0.83 (0.42, 1.63)	0.54 (0.27, 1.08)	0.085	0.124	0.81 (0.62, 1.07)	0.134	0.219
Tryptophan	1.00	0.62 (0.31, 1.25)	1.79 (0.78, 4.14)	0.222	0.301	1.43 (0.97, 2.11)	0.070	0.133
Histidine	1.00	1.49 (0.75, 2.93)	0.85 (0.44, 1.65)	0.644	0.723	0.81 (0.62, 1.07)	0.138	0.219
Non-essential amino acids								
Glutamic acid	1.00	2.96 (1.37, 6.39)	4.04 (1.79, 9.12)	0.001	0.006	1.89 (1.34, 2.67)	<0.001	0.006
Alanine	1.00	1.73 (0.82, 3.65)	3.41 (1.63, 7.14)	0.001	0.006	1.73 (1.23, 2.42)	0.001	0.006
Glutamine	1.00	0.66 (0.27, 1.62)	0.32 (0.12, 0.87)	0.017	0.057	0.65 (0.44, 0.97)	0.035	0.083
Glycine	1.00	0.51 (0.26, 1.00)	0.47 (0.26, 0.87)	0.018	0.057	0.73 (0.55, 0.96)	0.024	0.073
Tyrosine	1.00	1.82 (0.90, 3.66)	2.12 (1.03, 4.37)	0.044	0.084	1.23 (0.90, 1.68)	0.186	0.252
Serine	1.00	1.27 (0.70, 2.32)	1.81 (0.93, 3.52)	0.083	0.124	1.21 (0.91, 1.60)	0.186	0.252
Asparagine	1.00	1.20 (0.63, 2.29)	0.66 (0.32, 1.36)	0.299	0.379	0.86 (0.63, 1.18)	0.362	0.430
Arginine	1.00	0.95 (0.47, 1.90)	0.86 (0.46, 1.62)	0.647	0.723	0.92 (0.71, 1.20)	0.541	0.605
Proline	1.00	1.70 (0.83, 3.47)	1.00 (0.49, 2.07)	0.935	0.970	1.00 (0.76, 1.32)	0.997	0.997
Aspartic acid	1.00	0.67 (0.33, 1.38)	1.04 (0.43, 2.50)	0.970	0.970	1.05 (0.78, 1.41)	0.767	0.810

Prevalent cases were defined as those had HbA1c ≥ 6.5% (47.5 mmol/mol) but did not report to have diagnosed diabetes at baseline when blood samples were collected (1999–2004), while reported to have diagnosed diabetes during the follow-up (2006–2010). ^a^ Odds ratio (OR) with 95% confidence interval (CI) and *p* values were calculated by conditional logistic regression after adjustment for BMI, history of hypertension, smoking status, HDL-cholesterol, and triglycerides.

**Table 3:** Potential of amino acids for risk prediction of incident type 2 diabetes (*n* = 160).

Amino acids	C-Statistics ^a^		IDI	NRI
	AUC (95%CI)	*p* Value	*p* Value	*p* Value
Essential				
Valine	0.76 (0.70, 0.81)	0.34	0.03	0.01
Tryptophan	0.75 (0.70, 0.81)	0.51	0.05	0.09
Phenylalanine	0.76 (0.70, 0.81)	0.33	0.07	0.06
Leucine	0.75 (0.70, 0.80)	0.46	0.08	0.03
Isoleucine	0.75 (0.70, 0.80)	0.82	0.20	0.91
Histidine	0.75 (0.70, 0.80)	0.60	0.31	0.02
Methionine	0.75 (0.70, 0.80)	0.88	0.44	0.99
Lysine	0.75 (0.70, 0.80)	0.94	0.59	0.15
Threonine	0.75 (0.69, 0.80)	0.61	0.91	0.99
Non-essential				
Tyrosine	0.76 (0.71, 0.81)	0.21	0.02	0.01
Aspartic acid	0.75 (0.70, 0.81)	0.36	0.10	0.50
Glycine	0.75 (0.70, 0.81)	0.38	0.14	0.99
Alanine	0.75 (0.70, 0.80)	0.62	0.31	0.31
Glutamic acid	0.75 (0.70, 0.80)	0.76	0.38	0.37
Arginine	0.75 (0.69, 0.80)	0.92	0.69	0.50
Proline	0.75 (0.70, 0.80)	0.56	0.77	0.37
Serine	0.75 (0.69, 0.80)	0.87	0.80	0.58
Glutamine	0.75 (0.70, 0.80)	0.89	0.83	0.65
Asparagine	0.75 (0.69, 0.80)	0.08	0.99	0.65
Combinations				
Combination 1	0.76 (0.71, 0.82)	0.15	<0.01	<0.01
Combination 2	0.77 (0.72, 0.82)	0.12	<0.01	<0.01
Combination 3	0.77 (0.72, 0.82)	0.07	<0.01	<0.01
Combination 4	0.78 (0.73, 0.83)	0.04	<0.01	<0.01

IDI, integrated discrimination improvement; NRI, category-free net reclassification improvement. Combination 1: 6 amino acids associated with incident type 2 diabetes, including isoleucine, leucine, tryptophan, valine, glutamine, and tyrosine. Combination 2: 9 essential amino acids, including histidine, isoleucine, leucine, lysine, methionine, phenylalanine, threonine, tryptophan, and valine. Combination 3: 10 non-essential amino acids, including alanine, arginine, asparagine, aspartic acid, glutamic acid, glutamine, glycine, proline, serine, and tyrosine. Combination 4: all the 19 measure amino acids. ^a^ AUC increment of amino acids after adding in the basic model (AUC = 0.75, 95% CI [0.69, 0.80]) of BMI, history of hypertension, smoking, HDL-cholesterol, and triglycerides.

## Supplementary Materials

The following are available online at http://www.mdpi.com/2218-1989/9/1/14/s1, Table S1: Optimized MRM conditions for determination of 19 amino acids, Table S2: Method validation for determination of 19 amino acids, Table S3: Metabolite concentrations (µM, Mean ± SD) in controls stratified by fasting status, Table S4: Odds ratios (OR) with 95% confidence interval (CI) between amino acids and prevalent type 2 diabetes (*n* = 144) stratified by fasting status, Table S5: Odds ratios (OR) with 95% confidence interval (CI) between amino acids and incident type 2 diabetes (*n* = 160) stratified by fasting status.
