# Supplementary material for: Serum Amino Acids in Association with Prevalent and Incident Type 2 Diabetes in A Chinese Population
Source: Metabolites. 2019 Jan 14;9(1):14. doi: 10.3390/metabo9010014 (PMC6359471; doi:10.3390/metabo9010014)
Supplement: Supplementary file 1 [file metabolites-09-00014-s001.pdf]

Table S1. Optimized MRM conditions for determination of 19 amino acids

Amino acids	HMDB ID	R.T. (min)	Transition ion (m/z)	Fragmentor voltage (V)	CE (eV)	Internal standard	Calibration curve
Essential							
Histidine	HMDB0000177	15.11	156.1 -> 110.0	100	20	$^{13}C_6$, $^{15}N_3$-Histidine	Histidine
Isoleucine	HMDB0000172	9.11	132.1 -> 86.1	90	10	$^{13}C_6$, ^{15}N-Isoleucine	Isoleucine
Leucine	HMDB0000687	8.53	132.1 -> 86.1	90	10	$^{13}C_6$, ^{15}N-Leucine	Leucine
Lysine	HMDB0000182	15.38	147.1 -> 84.0	90	14	$^{13}C_6$, $^{15}N_2$-Lysine	Lysine
Methionine	HMDB0000696	10.01	150.1 -> 104.0	90	10	$^{13}C5$, ^{15}N-Methionine	Methionine
Phenylalanine	HMDB0000159	8.14	166.1 -> 120.1	90	14	$^{13}C_9$, ^{15}N-Phenylalanine	Phenylalanine
Threonine	HMDB0000167	12.87	120.1 -> 74.0	90	20	$^{13}C_4$, ^{15}N-Threonine	Threonine
Tryptophan	HMDB0000929	8.15	205.1 -> 146.0	70	18	$^{13}C_9$, ^{15}N-Phenylalanine	Tryptophan
Valine	HMDB0000883	10.79	118.1 -> 72.1	90	10	$^{13}C_5$, ^{15}N-Valine	Valine
Non-essential							
Alanine	HMDB0000161	11.97	90.1 -> 44.0	70	10	$^{13}C_3$, ^{15}N-Alanine	Alanine
Arginine	HMDB0000517	15.23	175.1 -> 70.0	90	20	$^{13}C_6$, $^{15}N_4$-Arginine	Arginine
Asparagine	HMDB0000168	14.15	133.1 -> 74.0	70	14	$^{13}C_2$, ^{15}N-Glycine	Asparagine
Aspartic acid	HMDB0000191	15.03	134.0 -> 74.0	90	15	$^{13}C_4$, ^{15}N-Aspartic acid	Aspartic acid
Glutamic acid	HMDB0000148	14.59	148.1 -> 84.0	90	14	$^{13}C_5$, ^{15}N-Glutamic acid	Glutamic acid
Glutamine	HMDB0000641	13.95	147.1 -> 84.0	70	16	$^{13}C_6$, $^{15}N_3$-Histidine	Glutamine
Glycine	HMDB0000123	13.30	76.0 -> 30.0	70	6	$^{13}C_2$, ^{15}N-Glycine	Glycine
Proline	HMDB0000162	10.79	116.1 -> 70.0	90	14	$^{13}C_5$, ^{15}N-Proline	Proline
Serine	HMDB0000187	14.00	106.1 -> 60.0	90	8	$^{13}C_3$, ^{15}N-Serine	Serine
Tyrosine	HMDB0000158	10.78	182.1 -> 136.0	90	16	$^{13}C_9$, ^{15}N-Tyrosine	Tyrosine

All the 19 metabolites were level 1 identifications according to Metabolomics Standards Initiative (MSI).

Table S2. Method validation for determination of 19 amino acids

Amino acids	LOD (µmol/L) S/N = 3	Linearity range (µmol/L) (R^2 >0.997)	Recovery (%)			Precision (RSD %)	
			Low	Medium	High	Intra-batch	Inter-batch
Essential							
Histidine	0.100	0.1-200	88	103	114	1.76	3.77
Isoleucine	0.030	0.1-200	97	100	112	0.80	2.02
Leucine	0.030	0.1-200	99	103	114	0.84	2.59
Lysine	0.003	0.1-200	93	109	111	1.68	3.52
Methionine	0.060	0.2-100	110	95	103	5.07	4.74
Phenylalanine	0.010	0.1-200	93	106	113	1.11	2.45
Threonine	0.200	0.2-200	105	101	112	2.67	5.32
Tryptophan	0.005	0.05-100	95	98	115	1.79	4.97
Valine	0.030	0.1-200	108	109	108	2.44	3.58
Non-essential							
Alanine	0.100	0.1-200	108	107	111	1.58	3.29
Arginine	0.003	0.1-100	84	96	106	1.71	4.81
Asparagine	0.015	0.2-100	89	96	109	2.24	5.14
Aspartic acid	0.800	0.8-200	107	91	107	3.30	6.78
Glutamic acid	0.015	0.1-200	106	111	116	1.63	3.32
Glutamine	0.009	0.2-400	97	113	112	2.21	5.30
Glycine	0.200	0.2-200	102	102	110	3.94	6.07
Proline	0.020	0.1-200	95	109	112	1.49	3.03
Serine	0.050	0.1-200	101	96	108	1.74	5.07
Tyrosine	0.190	0.2-200	92	92	100	5.24	5.82

Table S3. The associations between amino acids and incident type 2 diabetes (n = 160)

Amino acids	OR across tertiles (T) [a]					OR per SD increment [a]		
	T1	T2, OR (95% CI)	T3, OR (95% CI)	*P* for trend	FDR	OR (95% CI)	*P* value	FDR
Essential amino acids								
Valine	1.00	1.62 (0.81, 3.26)	2.68 (1.19, 6.04)	0.017	0.200	1.50 (1.09, 2.08)	0.014	0.133
Isoleucine	1.00	1.39 (0.72, 2.68)	2.20 (1.02, 4.73)	0.045	0.214	1.37 (0.99, 1.90)	0.054	0.205
Leucine	1.00	1.58 (0.80, 3.14)	1.85 (0.93, 3.68)	0.080	0.304	1.44 (1.06, 1.95)	0.021	0.133
Tryptophan	1.00	1.10 (0.56, 2.18)	1.96 (0.88, 4.36)	0.114	0.361	1.62 (1.11, 2.37)	0.012	0.133
Histidine	1.00	0.93 (0.45, 1.89)	0.61 (0.28, 1.31)	0.201	0.527	0.84 (0.62, 1.15)	0.290	0.409
Lysine	1.00	1.56 (0.79, 3.09)	1.51 (0.77, 2.97)	0.222	0.527	1.20 (0.91, 1.57)	0.202	0.344
Methionine	1.00	1.22 (0.63, 2.35)	1.48 (0.73, 2.98)	0.276	0.583	1.23 (0.92, 1.64)	0.166	0.344
Phenylalanine	1.00	0.93 (0.47, 1.84)	1.34 (0.69, 2.60)	0.393	0.622	1.19 (0.90, 1.57)	0.217	0.344
Threonine	1.00	0.64 (0.32, 1.27)	0.84 (0.41, 1.72)	0.608	0.735	1.06 (0.80, 1.40)	0.689	0.873
Non-essential amino acids								
Glutamic acid	1.00	1.53 (0.76, 3.04)	2.55 (1.15, 5.64)	0.021	0.200	1.31 (0.95, 1.80)	0.099	0.314
Tyrosine	1.00	1.13 (0.57, 2.26)	2.26 (1.08, 4.59)	0.033	0.209	1.35 (1.00, 1.82)	0.046	0.205
Glycine	1.00	1.13 (0.53, 2.37)	0.70 (0.31, 1.54)	0.322	0.608	0.80 (0.59, 1.09)	0.154	0.344
Alanine	1.00	1.30 (0.62, 2.70)	1.41 (0.69, 2.90)	0.352	0.608	1.17 (0.87, 1.56)	0.301	0.409
Serine	1.00	1.05 (0.53, 2.10)	0.75 (0.36, 1.60)	0.429	0.627	0.96 (0.68, 1.34)	0.808	0.903
Asparagine	1.00	0.43 (0.19, 0.93)	1.12 (0.50, 2.55)	0.616	0.735	0.99 (0.72, 1.37)	0.963	0.995
Proline	1.00	1.24 (0.61, 2.51)	0.84 (0.42, 1.70)	0.619	0.735	0.96 (0.71, 1.28)	0.758	0.900
Glutamine	1.00	0.75 (0.26, 2.14)	0.83 (0.26, 2.61)	0.858	0.920	0.67 (0.38, 1.19)	0.170	0.344
Arginine	1.00	0.59 (0.29, 1.18)	0.97 (0.50, 1.88)	0.903	0.920	1.00 (0.77, 1.30)	0.995	0.995
Aspartic acid	1.00	0.90 (0.40, 2.04)	0.95 (0.39, 2.29)	0.920	0.920	1.26 (0.88, 1.81)	0.202	0.344

Incident cases were defined as those had HbA1c <6.5% (47.5 mmol/mol) at baseline when blood samples were collected (1999-2004), while reported to have diagnosed diabetes during the follow-up (2006-2010).

[a] Odds ratio (OR) with 95% confidence interval (CI) and P values were calculated by conditional logistic regression after adjustment for BMI, history of hypertension, smoking status, HDL-cholesterol, and triglycerides.

Table S4. Metabolite concentrations (μM, Mean ± SD) in controls stratified by fasting status

Amino acids	Non-fasting (n = 228)	Fasting (n = 76)	*P* [a]
Essential			
Histidine	84.57 ± 9.44	86.68 ± 9.97	0.108
Isoleucine	74.31 ± 20.28	67.91 ± 13.06	0.002
Leucine	146.36 ± 32.65	143.62 ± 23.02	0.423
Lysine	442.13 ± 78.73	442.93 ± 62.04	0.928
Methionine	29.89 ± 8.27	30.67 ± 5.27	0.340
Phenylalanine	92.58 ± 15.66	87.53 ± 11.57	0.003
Threonine	133.68 ± 26.01	137.94 ± 24.91	0.204
Tryptophan	53.32 ± 9.53	52.20 ± 7.93	0.314
Valine	348.04 ± 58.60	338.60 ± 49.99	0.175
Non-essential			
Alanine	474.59 ± 86.98	427.80 ± 82.24	<0.001
Arginine	110.44 ± 23.78	97.64 ± 18.18	<0.001
Asparagine	62.14 ± 12.13	61.99 ± 10.97	0.921
Aspartic acid	2.17 ± 0.86	2.19 ± 0.85	0.914
Glutamic acid	129.82 ± 40.43	136.79 ± 46.90	0.248
Glutamine	266.27 ± 58.54	261.22 ± 56.74	0.505
Glycine	276.81 ± 51.86	285.00 ± 59.88	0.288
Proline	134.31 ± 38.28	113.85 ± 38.96	<0.001
Serine	159.57 ± 25.17	165.00 ± 31.72	0.178
Tyrosine	75.72 ± 17.03	71.01 ± 12.95	0.013

[a] *P* values were calculated by the Student *t* test.

Table S5. Odds ratios (OR) with 95% confidence interval (CI) between amino acids and prevalent type 2 diabetes (n = 144) stratified by fasting status [a]

Amino acids	Fasting status	OR across tertiles					OR per SD increment		
		T1	T2, OR (95% CI)	T3, OR (95% CI)	*P* for trend	*P* for interaction	OR (95% CI)	*P*	*P* for interaction
Essential									
Histidine	Non-fasting	1.00	1.08 (0.52, 2.23)	0.67 (0.32, 1.40)	0.296	0.788	0.84 (0.62, 1.13)	0.256	0.415
	Fasting	1.00	1.45 (0.42, 5.08)	0.98 (0.30, 3.16)	0.909		0.66 (0.39, 1.11)	0.116	
Isoleucine	Non-fasting	1.00	2.76 (1.26, 6.01)	2.75 (1.26, 5.99)	0.014	0.586	1.30 (0.96, 1.74)	0.089	0.351
	Fasting	1.00	2.30 (0.74, 7.12)	1.94 (0.54, 7.03)	0.257		2.01 (0.92, 4.39)	0.079	
Leucine	Non-fasting	1.00	2.51 (1.19, 5.28)	2.05 (0.97, 4.32)	0.057	0.708	1.42 (1.05, 1.92)	0.023	0.580
	Fasting	1.00	2.82 (0.84, 9.40)	3.26 (0.91, 11.66)	0.073		1.78 (0.89, 3.53)	0.102	
Lysine	Non-fasting	1.00	0.81 (0.39, 1.68)	1.77 (0.85, 3.68)	0.135	0.620	1.30 (0.96, 1.76)	0.088	0.648
	Fasting	1.00	1.29 (0.38, 4.38)	3.07 (0.86, 11.04)	0.075		1.65 (0.90, 3.00)	0.104	
Methionine	Non-fasting	1.00	2.79 (1.28, 6.11)	1.74 (0.85, 3.57)	0.125	0.784	1.21 (0.92, 1.59)	0.177	0.831
	Fasting	1.00	1.79 (0.49, 6.56)	2.10 (0.50, 8.90)	0.340		1.22 (0.55, 2.66)	0.626	
Phenylalanine	Non-fasting	1.00	2.10 (0.96, 4.61)	2.34 (1.11, 4.93)	0.030	0.018	1.34 (0.99, 1.80)	0.057	0.259
	Fasting	1.00	3.00 (0.99, 9.04)	3.81 (0.94, 15.47)	0.031		1.88 (0.98, 3.60)	0.058	
Threonine	Non-fasting	1.00	0.61 (0.29, 1.27)	0.64 (0.30, 1.36)	0.251	0.906	0.83 (0.61, 1.26)	0.229	0.587
	Fasting	1.00	1.08 (0.34, 3.46)	0.83 (0.22, 2.48)	0.608		0.90 (0.56, 1.47)	0.683	
Tryptophan	Non-fasting	1.00	0.60 (0.28, 1.29)	1.39 (0.66, 2.94)	0.320	0.752	1.18 (0.89, 1.58)	0.250	0.857
	Fasting	1.00	0.45 (0.14, 1.43)	1.29 (0.40, 4.18)	0.734		1.28 (0.73, 2.27)	0.391	
Valine	Non-fasting	1.00	3.76 (1.75, 8.08)	2.49 (1.14, 5.42)	0.022	0.479	1.54 (1.11, 2.14)	0.010	0.309
	Fasting	1.00	1.63 (0.49, 5.38)	4.68 (1.23, 17.89)	0.023		2.23 (1.18, 4.23)	0.014	
Non-essential									
Alanine	Non-fasting	1.00	1.28 (0.57, 2.88)	3.55 (1.60, 7.89)	0.001	0.937	2.00 (1.37, 2.92)	<0.001	0.345
	Fasting	1.00	2.53 (0.85, 7.50)	2.94 (0.73, 11.82)	0.076		1.42 (0.81, 2.49)	0.216	
Arginine	Non-fasting	1.00	0.99 (0.47, 2.11)	0.77 (0.37, 1.60)	0.464	0.144	0.91 (0.68, 1.21)	0.519	0.107
	Fasting	1.00	0.97 (0.31, 3.02)	2.88 (0.8, 10.44)	0.142		1.72 (0.95, 3.12)	0.072	
Asparagine	Non-fasting	1.00	0.91 (0.43, 1.90)	0.84 (0.41, 1.71)	0.622	0.817	0.93 (0.70, 1.23)	0.600	0.509
	Fasting	1.00	2.84 (0.84, 9.56)	0.67 (0.19, 2.41)	0.677		0.80 (0.46, 1.38)	0.421	
Aspartic acid	Non-fasting	1.00	1.06 (0.51, 2.21)	1.09 (0.51, 2.35)	0.821	0.719	1.13 (0.81, 1.56)	0.468	0.925
	Fasting	1.00	0.40 (0.12, 1.35)	2.07 (0.62, 6.93)	0.322		1.18 (0.76, 1.84)	0.466	
Glutamic acid	Non-fasting	1.00	2.11 (1.04, 4.31)	2.84 (1.25, 6.44)	0.009	0.699	1.78 (1.25, 2.54)	0.001	0.400

	Fasting	1.00	3.21 (0.84, 12.28)	2.30 (0.72, 7.37)	0.214		1.34 (0.82, 2.18)	0.241	
Glutamine	Non-fasting	1.00	1.10 (0.52, 2.34)	0.44 (0.21, 0.93)	0.028	0.372	0.76 (0.56, 1.03)	0.082	0.678
	Fasting	1.00	0.65 (0.20, 2.04)	0.86 (0.26, 2.77)	0.774		0.93 (0.57, 1.50)	0.755	
Glycine	Non-fasting	1.00	0.50 (0.23, 1.05)	0.41 (0.19, 0.87)	0.020	0.594	0.67 (0.48, 0.94)	0.020	0.709
	Fasting	1.00	0.38 (0.12, 1.24)	0.33 (0.10, 1.11)	0.067		0.64 (0.35, 1.16)	0.142	
Proline	Non-fasting	1.00	1.21 (0.54, 2.69)	0.90 (0.40, 2.00)	0.727	0.190	1.01 (0.73, 1.40)	0.935	0.503
	Fasting	1.00	1.42 (0.81, 7.29)	2.38 (0.53, 10.66)	0.109		1.33 (0.81, 2.20)	0.256	
Serine	Non-fasting	1.00	1.18 (0.56, 2.47)	1.62 (0.77, 3.40)	0.203	0.427	1.17 (0.86, 1.60)	0.306	0.790
	Fasting	1.00	2.35 (0.69, 7.99)	3.18 (0.93, 10.92)	0.066		1.31 (0.79, 2.15)	0.295	
Tyrosine	Non-fasting	1.00	1.54 (0.73, 3.28)	1.87 (0.90, 3.92)	0.100	0.591	1.12 (0.84, 1.49)	0.447	0.139
	Fasting	1.00	1.86 (0.60, 5.71)	2.42 (0.70, 8.40)	0.148		1.74 (0.93, 3.25)	0.083	

[a] OR (95%CI) and *P* values were calculated by logistic regression after adjustment for age, sex, BMI, history of hypertension, smoking status, HDL-cholesterol, and triglycerides.

Table S6. Odds ratios (OR) with 95% confidence interval (CI) between amino acids and incident type 2 diabetes (n = 160) stratified by fasting status [a]

Amino acids	Fasting status	OR across tertiles					OR per SD increment		
		T1	T2, OR (95% CI)	T3, OR (95% CI)	*P* for trend	*P* for interaction	OR (95% CI)	*P*	*P* for interaction
Essential									
Histidine	Non-fasting	1.00	0.97 (0.49, 1.90)	0.90 (0.45, 1.81)	0.775	0.085	0.93 (0.70, 1.23)	0.626	0.489
	Fasting	1.00	0.46 (0.11, 1.92)	0.20 (0.05, 0.84)	0.027		0.76 (0.45, 1.28)	0.300	
Isoleucine	Non-fasting	1.00	1.56 (0.77, 3.16)	1.58 (0.77, 3.25)	0.219	0.785	1.20 (0.90, 1.60)	0.212	0.460
	Fasting	1.00	0.58 (0.15, 2.18)	4.44 (0.65, 30.55)	0.256		1.93 (0.79, 4.70)	0.150	
Leucine	Non-fasting	1.00	1.90 (0.94, 3.83)	1.39 (0.70, 2.78)	0.353	0.187	1.25 (0.94, 1.65)	0.127	0.265
	Fasting	1.00	1.08 (0.28, 4.17)	6.50 (0.97, 43.38)	0.070		2.06 (0.85, 5.00)	0.109	
Lysine	Non-fasting	1.00	1.74 (0.86, 3.52)	1.25 (0.64, 2.44)	0.525	0.992	1.10 (0.85, 1.43)	0.464	0.632
	Fasting	1.00	0.49 (0.13, 1.87)	1.20 (0.28, 5.17)	0.773		1.28 (0.62, 2.62)	0.500	
Methionine	Non-fasting	1.00	1.00 (0.50, 2.00)	0.97 (0.50, 1.90)	0.932	0.707	1.09 (0.85, 1.42)	0.495	0.729
	Fasting	1.00	1.77 (0.44, 7.07)	1.33 (0.30, 5.96)	0.763		1.42 (0.62, 3.26)	0.405	
Phenylalanine	Non-fasting	1.00	1.14 (0.57, 2.31)	1.87 (0.94, 3.72)	0.073	0.724	1.29 (0.97, 1.70)	0.076	0.703
	Fasting	1.00	0.87 (0.25, 3.09)	1.70 (0.37, 7.86)	0.551		1.59 (0.71, 3.58)	0.262	
Threonine	Non-fasting	1.00	0.57 (0.28, 1.16)	0.95 (0.47, 1.89)	0.899	0.940	1.07 (0.81, 1.41)	0.650	0.554
	Fasting	1.00	0.31 (0.07, 1.36)	0.82 (0.20, 3.43)	0.928		0.96 (0.53, 1.72)	0.889	
Tryptophan	Non-fasting	1.00	1.05 (0.52, 2.10)	1.68 (0.84, 3.34)	0.138	0.494	1.39 (1.04, 1.85)	0.025	0.329
	Fasting	1.00	0.76 (0.21, 2.74)	0.99 (0.25, 3.91)	0.944		0.97 (0.51, 1.84)	0.926	
Valine	Non-fasting	1.00	1.68 (0.83, 3.40)	2.01 (0.98, 4.15)	0.059	0.841	1.36 (1.00, 1.85)	0.050	0.455
	Fasting	1.00	0.92 (0.27, 3.18)	2.5 (0.47, 13.42)	0.364		1.72 (0.84, 3.52)	0.135	
Non-essential									
Alanine	Non-fasting	1.00	1.29 (0.63, 2.65)	1.19 (0.58, 2.46)	0.665	0.242	1.10 (0.83, 1.46)	0.507	0.228
	Fasting	1.00	2.51 (0.75, 8.38)	1.59 (0.31, 8.30)	0.330		1.50 (0.72, 3.13)	0.278	
Arginine	Non-fasting	1.00	0.68 (0.33, 1.40)	0.96 (0.48, 1.93)	0.986	0.661	1.01 (0.77, 1.33)	0.943	0.467
	Fasting	1.00	0.53 (0.16, 1.76)	2.38 (0.5, 11.46)	0.521		1.40 (0.75, 2.62)	0.287	
Asparagine	Non-fasting	1.00	0.69 (0.34, 1.40)	1.43 (0.71, 2.89)	0.272	0.123	1.07 (0.82, 1.41)	0.615	0.393
	Fasting	1.00	0.23 (0.06, 0.89)	0.45 (0.11, 1.82)	0.193		0.79 (0.44, 1.42)	0.437	
Aspartic acid	Non-fasting	1.00	1.31 (0.66, 2.61)	0.97 (0.49, 1.95)	0.922	0.360	1.13 (0.82, 1.54)	0.465	0.444
	Fasting	1.00	0.43 (0.11, 1.76)	2.79 (0.71, 10.92)	0.216		1.68 (0.94, 2.99)	0.079	
Glutamic acid	Non-fasting	1.00	1.13 (0.57, 2.24)	1.61 (0.81, 3.21)	0.177	0.556	1.13 (0.84, 1.51)	0.430	0.936

	Fasting	1.00	1.04 (0.27, 4.00)	1.19 (0.27, 5.25)	0.818		1.29 (0.72, 2.33)	0.396	
Glutamine	Non-fasting	1.00	1.04 (0.52, 2.07)	1.23 (0.61, 2.48)	0.560	0.468	1.08 (0.81, 1.44)	0.587	0.135
	Fasting	1.00	1.24 (0.34, 4.55)	0.53 (0.14, 2.06)	0.398		0.58 (0.31, 1.06)	0.076	
Glycine	Non-fasting	1.00	1.16 (0.58, 2.34)	0.73 (0.36, 1.46)	0.367	0.839	0.84 (0.63, 1.12)	0.236	0.618
	Fasting	1.00	1.63 (0.41, 6.50)	0.84 (0.21, 3.31)	0.760		0.73 (0.40, 1.34)	0.314	
Proline	Non-fasting	1.00	1.57 (0.77, 3.24)	0.98 (0.48, 2.00)	0.826	0.637	0.95 (0.72, 1.26)	0.716	0.617
	Fasting	1.00	1.81 (0.53, 6.21)	1.57 (0.30, 8.23)	0.444		1.35 (0.67, 2.73)	0.406	
Serine	Non-fasting	1.00	1.23 (0.61, 2.48)	0.82 (0.41, 1.64)	0.578	0.467	1.02 (0.74, 1.40)	0.909	0.401
	Fasting	1.00	0.21 (0.05, 0.95)	0.27 (0.06, 1.26)	0.139		0.70 (0.40, 1.23)	0.217	
Tyrosine	Non-fasting	1.00	1.01 (0.50, 2.07)	2.15 (1.08, 4.30)	0.027	0.597	1.35 (1.02, 1.78)	0.035	0.851
	Fasting	1.00	1.38 (0.41, 4.64)	1.54 (0.34, 7.03)	0.530		1.71 (0.80, 3.66)	0.163	

[a] OR (95%CI) and *P* values were calculated by logistic regression after adjustment for age, sex, BMI, history of hypertension, smoking status, HDL-cholesterol, and triglycerides.
